# Effect of TIM-3 Blockade on the Immunophenotype and Cytokine Profile of Murine Uterine NK Cells

**DOI:** 10.1371/journal.pone.0123439

**Published:** 2015-04-21

**Authors:** Sudipta Tripathi, Lola Chabtini, Pranal J. Dakle, Brian Smith, Hisaya Akiba, Hideo Yagita, Indira Guleria

**Affiliations:** 1 Children’s Hospital Boston, Renal Division, Harvard Medical School, Boston, Massachusetts, United States of America; 2 Department of Immunology, Juntendo University, Tokyo, Japan; Xavier Bichat Medical School, FRANCE

## Abstract

NK cells are the most abundant lymphocyte population in the feto-maternal interface during gestation. The uterine NK cells (uNK) are transient, have a unique immunophenotype and produce a number of cytokines. These cytokines play an important role in establishment and maintenance of vascular remodeling and tolerance associated with successful pregnancy. The uNK cells also express TIM-3 during gestation and blockade of TIM-3 expression results in fetal loss in mice. In this study we determined the effect of TIM-3 blockade on uNK cells. Specifically we observed surface receptor phenotype and cytokine production by uNK cells following TIM-3 blockade. Our results show that TIM-3 plays a role in regulating the uNK cells and contributes to the maintenance of tolerance at the feto-maternal interface.

## Introduction

NK cells are the most abundant lymphocyte population, approximately 70%, in the uterus during early gestation in humans. In mice, uterine NK (uNK) cells start to accumulate after gestation day (GD) 4, peak in number during mid gestation (GD 10–12), decline during the late stages and disappear completely postpartum [1]. Uterine NK cells are known to play a critical role in the establishment and maintenance of pregnancy in mice and are necessary for the vascular remodeling that occurs during pregnancy [2]. Uterine NK cells in mice also differ from the peripheral/circulating NK cells (splenic NK cells) in their unique surface phenotype and functional plasticity [3] and play a role in modulating tolerance at the feto-maternal interface (FMI) [4,5].

The T-cell immunoglobulin mucin -3 (TIM-3) is a type-1 glycoprotein that is expressed on the cells of both innate and adaptive immune system. TIM-3 is a novel costimulatory molecule of the TIM family, and is involved in regulating the T cell responses by interacting with its ligand galectin-9 [6]. TIM-3/Galectin-9 signaling is also involved in regulating tolerance to allograft in murine models of transplantation [7]. Dysregulation of TIM-3 in innate immune cells is associated with pathogenesis and exacerbation of disease in chronic viral infections [8,9] and tumors [10,11] but the underlying mechanisms are yet to be determined. TIM-3 also plays a role in the maintenance of tolerance to the fetus. We have shown previously that blockade of TIM-3 results in abrogation of phagocytic activity of the uterine macrophages and accumulation of apoptotic cells at the feto-maternal interface leading to fetal loss [12]. Abnormal TIM-3 expression is associated with fetal loss in humans too [13].

TIM-3 expression on NK cells is reported to regulate their cytotoxicity [14], cytokine production [15] and also regulate the immune response [16,17]. Given the fact that NK cells are the most abundant lymphocyte population at the FMI and play a major role in regulating tolerance at the FMI we aimed to explore the effect of TIM-3 blockade on uNK cells. Further, to understand the role of TIM-3 in regulation of tolerance at the FMI, we studied the effect of TIM-3 blockade on uNK cells in a mouse model of allogeneic pregnancy.

In the current study we show that blockade of TIM-3 changes both the phenotype and functionality of the uNK cells at the FMI. Following TIM-3 blockade, expression of the receptor repertoire on uNK cells was altered and production of various cytokine by the uNK cells was decreased resulting in dysregulation of the fine balance between immunity and tolerance at the FMI contributing to fetal loss.

## Materials and Methods

### Mice

CBA/CaJ, C57BL/6 and B6.Cg-Tg(TcraTcrb)425Cbn/J (OT II) mice were purchased from the Jackson Laboratories and maintained in the Boston Children’s Hospital animal facility according to the institutional guidelines. 6 to 7 weeks old CBA/CaJ females were mated with C57BL/6 males and vaginal plugs were monitored everyday. For certain experiments C57BL/6 females were mated with CBA males and CBA females were syngeneically mated with CBA males. The day of visualization of the plug was designated as gestation day (GD) 0.5. Pregnant mice were randomly divided into two groups, control and treated, for some of the experiments. The treated group were injected i.p with anti TIM-3 mAb (clone RMT3-23, BioXCell) at doses 500μg, 250μg and 250μg at GD 6.5, 8.5 and 10.5 respectively [12]. The control group received phosphate buffered saline.

### Ethics Statement

All mice were cared for in accordance with Boston Children’s Hospital institutional guidelines. All mouse experiments were approved by the Institutional Animal Care and Use Committee of Children's Hospital Boston.

### Lymphocyte isolation

Pregnant mice were sacrificed between GD10.5 to 12.5 and uteri were dissected free from the mesometrium and removed by cuts at the ovaries and the cervix. The uteri were further dissected to remove the fetal and placental tissue. The placentae containing the decidua basalis were collected separately for further processing for either RNA isolation or homogenate preparation. The uteri wall tissue comprising the mesometrial decidua were washed in HBSS, minced and subjected to enzymatic digestion for 40 min at 37°C in HBSS containing 200U/ml Hyaluronidase, 1mg/ml collagenase, 0.2mg/ml DNase and 1mg/ml BSA. The digested tissue was then washed and incubated in enzyme free HBSS for another 15 min at 37°C followed by filtration through a 70μM cell strainer. These cells were then stained with antibodies for flow cyotmetry analysis of cell surface receptors and intracellular cytokines.

### Flow cytometry and intracellular staining

The uterine lymphocytes were pretreated with Fc block anti CD16 (clone 24G.2) for 10 min at 4°C and washed with FACS buffer (HBSS, 10% FBS). The following fluorochrome labeled antibodies against CD45 (30-F11), CD11c (N418), TIM-3 (B8.2C12), Ly6C (HK1.4), Ly6G (1A8), CD3 (17A2), were purchased from BioLegend, eBiosciences or BD Biosciences. Biotinylated DBA was purchased from Sigma-Aldrich. For intracellular cytokine staining, uterine lymphocytes were stimulated with PMA/Ionomycin for 4h at 37°C in presence of Golgistop. Following stimulation, cells were stained for surface markers and then permeabilized for 30 min at 4°C using the Fix-Perm kit from BD Biosciences and stained for various intracellular cytokines and Granzyme B. The cells were then washed with FACS buffer and acquired on a 3-laser, 8-colour BD FACS Canto II cytometer. The data were analyzed using FlowJo (Tree Star)

### Cell culture

Uterine lymphocytes were flow sorted for uNK cells (NK1.1^-^DX5^-^DBA^+^) and cultured for 5–7 days at 37°C in a humidified CO_2_ incubator in presence of various cytokine combinations. The cytokine concentrations used were GMCSF (20ng/ml), IL-2 (2ng/ml), IL-12 (2ng/ml) and IL-15 (50ng/ml) respectively. At the end of incubation cells were analyzed by flow cytometry to determine the percentage of uNK and uMDSC (uterine myeloid derived suppressor cells) population.

For the MDSC suppression assay, uMDSC (CD11b^+^ Ly6C^+^ Ly6G^+^) were flow sorted and co-cultured at a ratio of 1:5:1 with OVA_323-339_ specific CD4^+^ T cells from OT-II mouse and CD90^-^ allogeneic APC from C57BL/6 mouse isolated using micro beads from Miltenyi. The cells were incubated for 48-72h at 37°C in a humidified CO_2_ incubator and CD4^+^ T cell proliferation was determined by CFSE dilution.

### Quantification of cytokines using Luminex and ELISA

Cytokine concentrations in serum, culture supernatant and placental homogenate were determined using either ELISA kits or LUMINEX. All ELISA were performed according to the kit manufacturer’s protocol (R&D systems). Luminex assay was performed using preconfigured 21-plex cytokine bead based immunoassay according to manufacturer’s protocol (Millipore)

### Quantification of chemokines using real time PCR

Total RNA was extracted from placentae and/or uteri using TRIZOL (Invitrogen), followed by cDNA synthesis using SuperScript III First-Strand Synthesis SuperMix (Invitrogen). mRNA expression of various chemokines was determined using TaqMan gene expression assay. Target gene values were calculated relative to GAPDH expression.

### Statistics

Unpaired two-tailed t-test was used to analyze the statistical significance between two groups. Statistical significance between multiple groups was determined using Kruskal-Wallis and/or two way ANOVA analysis.

## Results

### Phenotypic characterization of uNK cells at the feto-maternal interface in CBA/CaJ mouse

**Fig 1:**
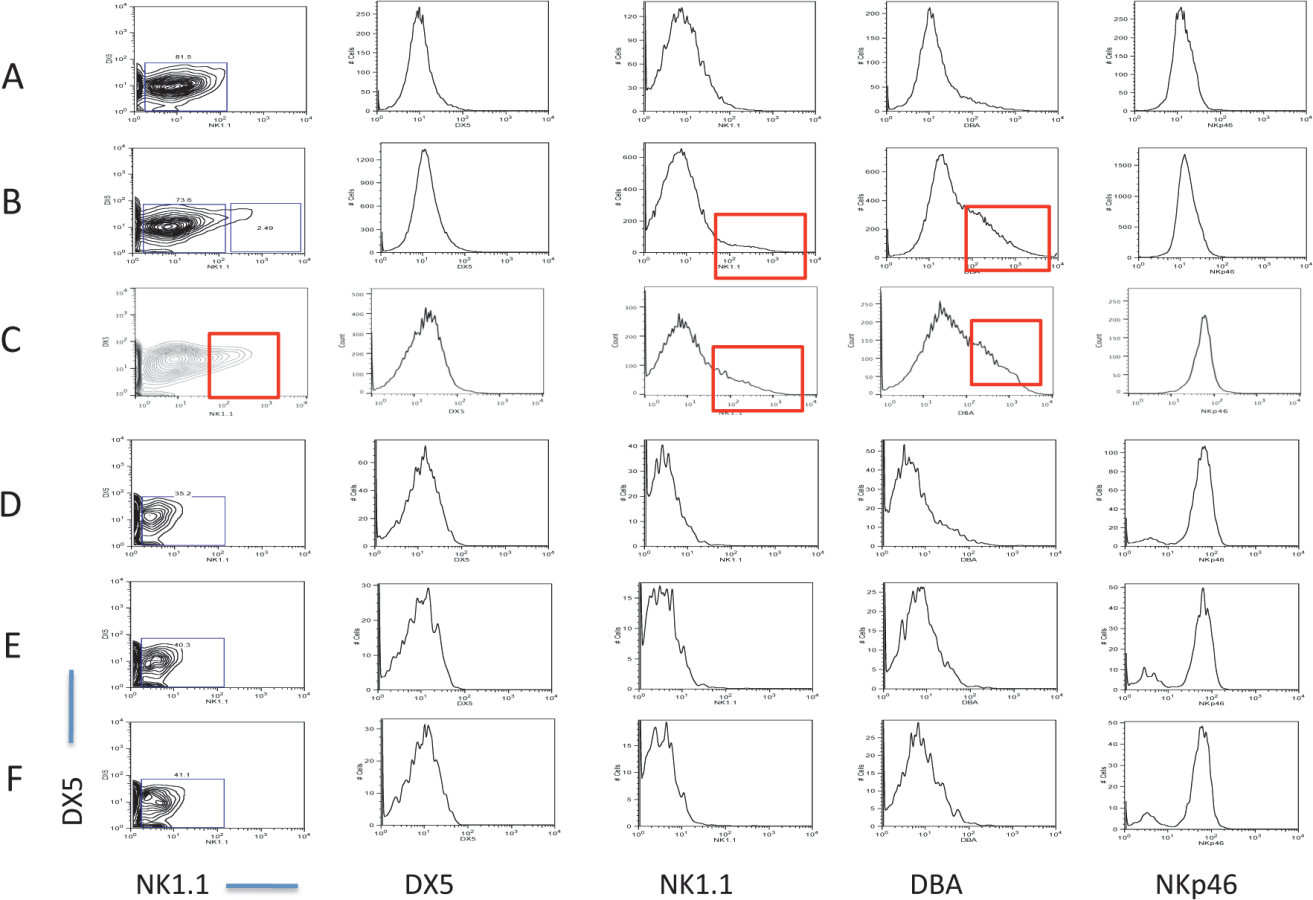
Expression of NK cell markers in the uNK and pNK population from non-pregnant and pregnant mice. Expression of NK1.1, DX5, DBA and NKp46 were determined on CD3^-^CD122^+^ cells isolated from the uteri of non-pregnant (A) syngeneically mated pregnant (B) and allogeneically mated (C) pregnant CBA mice. Expression of NK1.1, DX5, DBA and NKp46 on CD3^-^CD122^+^ cells from spleen of non-pregnant CBA mouse (D), syngeneically mated (E) and allogeneically mated (F) pregnant CBA mouse. Red-boxed areas denote uNK populations positive for NK1.1 and DBA in allogeneically mated pregnant CBA mouse (C) and DBA positive uNK population in syngeneically mated pregnant CBA mouse (B). These positive populations are absent in the splenic NK cell population in the same mouse. Data is representative of at least 3 experiments.

We compared the expression of several NK cell surface receptors in non-pregnant, and pregnant CBA/CaJ mouse strain. We used two different models of pregnancy, syngeneic (CBA female x CBA male) and allogeneic (CBA female x C57BL/6 male). The uterine lymphocytes were isolated between GD 10.5 and 12.5 and stained for various NK cell markers and analyzed using a flow cytometer. We determined the expression of NK1.1, DX5, DBA and NKp46 (NCR1) on CD3^-^CD122^+^ cells isolated from the spleen and uteri. Fig 1 shows the expression of the above-mentioned four NK cell markers on the spleens and uteri from non-pregnant and pregnant mice. Both the pNK and uNK populations from non-pregnant CBA mouse were negative for NK1.1 and DX5 expression. The pNK cells from non-pregnant mice were also negative for DBA expression and a very small percentage of uNK cells were positive for DBA. NKp46 expression level was high on pNK cells and low on uNK cells [Fig 1A and 1D]. Expression of the above mentioned four markers in the uNK and pNK cells of syngeneically mated pregnant mice are shown in Fig 1B and 1E and the same for allogeneically mated pregnant mice are shown in Fig 1C and 1F. In both the syngeneically and allogeneically mated pregnant CBA mice the expression of DBA is increased in the uNK cells [Fig 1B and 1C].

We also observed an unusual phenomenon in the uNK cells of the syngeneically mated pregnant CBA mice. A small but significant increase (~ 2.5% of the CD3^-^CD122^+^ cells) in the NK1.1 expression was observed in the uNK population (Fig 1B). We did not observe any NK1.1 expression in splenic pNK population from the same mouse [Fig 1E]. This observation was very unusual, as NK cells from CBA mouse strain do not react to the anti NK1.1 monoclonal antibody PK136. This is due to allelic divergence of the *Nkrp1b/c* gene in the CBA mouse strain that confers NK1.1 reactivity [18]. Interestingly, we observed an additional increase in NK1.1 reactivity in the pregnant CBA mouse that was allogeneically mated to C57BL/6 male. Not only did the uNK cells from allogeneically mated pregnant CBA mice expressed NK1.1 but the expression level was also significantly higher (~20% of the CD3^-^CD122^+^ cells) than that of the syngeneically mated CBA mice [Fig 1C]. We did not observe this phenomenon in the non-pregnant mice [Fig 1A and 1D].

### Selectively altered cytokine and chemokine milieu in the placenta following treatment with RMT3-23

**Fig 2:**
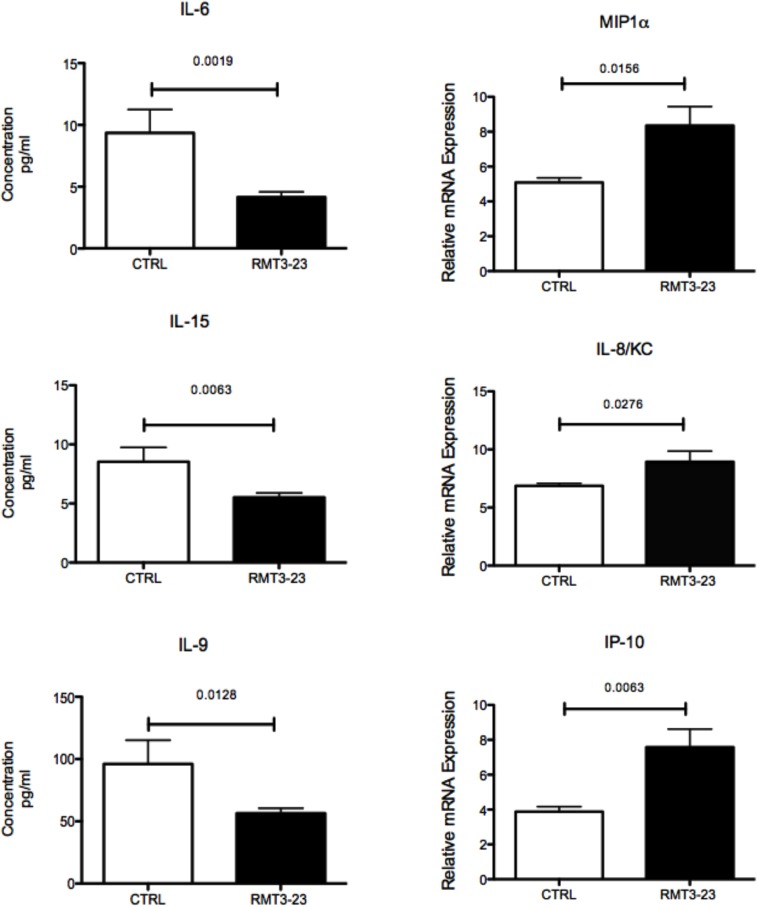
Effect of RMT3-23 treatment on the cytokine and chemokine milieu in the placenta and on the uNK cell population size of pregnant CBA mice between G.D 10.5 to 12.5 that were allogeneically mated to C57BL/6 males. A significant decrease in IL-6, IL-15 and IL-9 concentrations and a significant increase in MIP1α, IL-8/KC and IP-10 mRNA expression is observed in the placental homogenate from mice treated with RMT3-23 in comparison to the control group. Data are presented as mean± SEM. Statistical analysis was done using unpaired t test. However, there was no significant change in the number of uNK cells (CD3- CD122+ NK1.1- and DX5-) between control and treated groups following treatment with RMT3-23. Data are presented as mean± SEM and are representative of at least 3 experiments. Statistical analysis was done by one-way analysis of variance (ANOVA) using Kruskal-Wallis test.

We determined if TIM-3 blockade significantly changes the cytokine and chemokine milieu at the FMI that affects the uNK cells. Allogeneically mated (CBA female x C57BL/6 male) female CBA mice treated with RMT3-23 (anti TIM-3) showed a decrease in the production of IL-6 (p = 0.0019), IL-15 (p = 0.0063) and IL-9 (p = 0.0128) in the placenta between G.D 10.5 and 12.5 [Fig 2]. The chemokine production in the placentae was also altered and a significant increase in the production of MIP1α (p = 0.0156), KC (murine equivalent of IL-8, p = 0.0276) and IP-10 (p = 0.0063) was observed in the placentae of RMT3-23 treated mice at G.D 10.5 [Fig 2].

### Effect of TIM-3 blockade on uterine NK cell population during gestation

**Fig 3:**
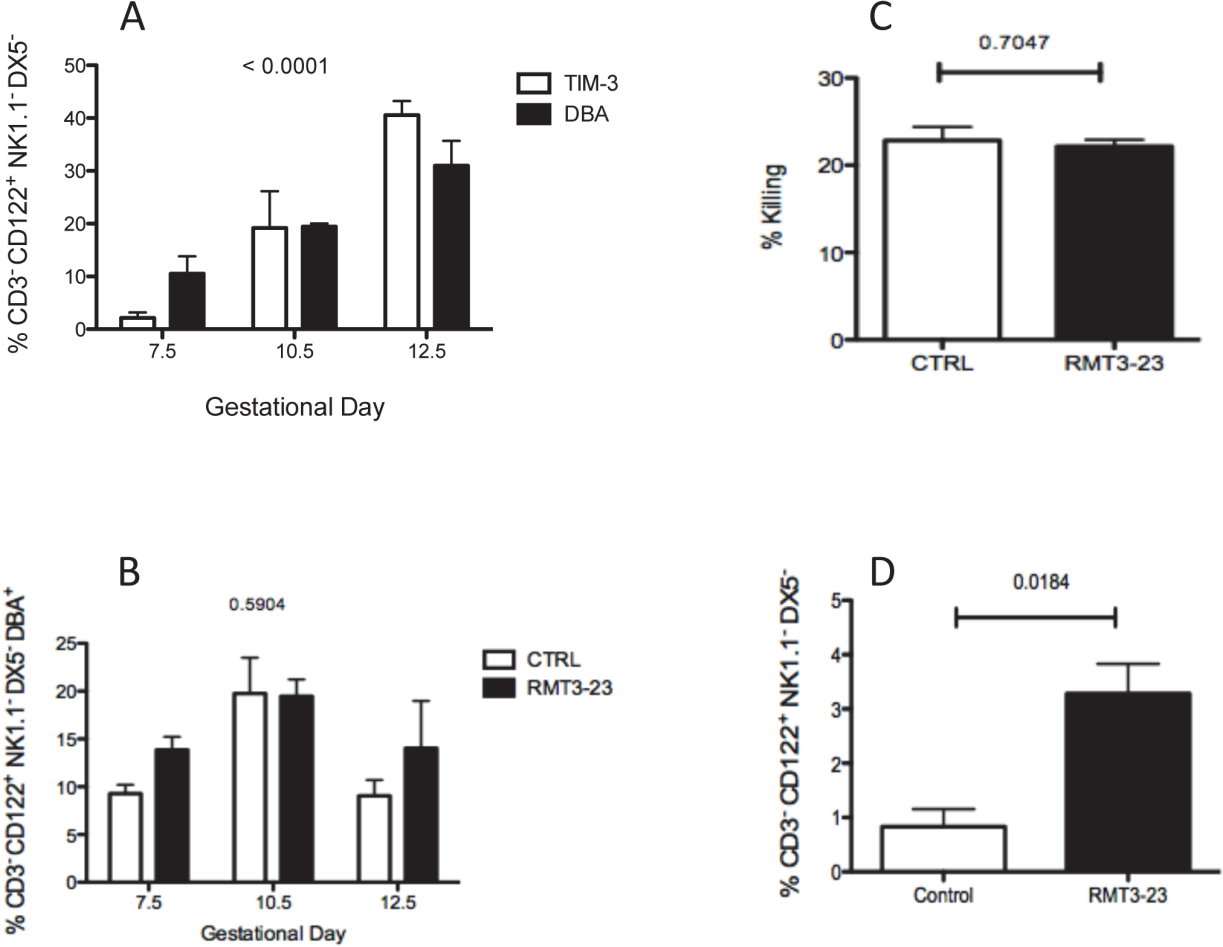
A. Expression of TIM-3 and DBA on uNK cells during gestation. A steady and similar increase in the expression of both TIM-3 and DBA is observed between GD 7.5 and 12.5, the time period when the uNK cells are most abundant in the uterus. TIM-3 and DBA expression were determined on the CD3^-^ CD122^+^ NK1.1^-^ DX5^-^ cells isolated from the uterus of pregnant CBA mice allogeneically mated with C57BL/6 males (n = 8). Data are presented as mean± SEM and are representative of at least 3 experiments. Data show the percentage of CD3- CD122+ NK1.1- DX5- cells expressing DBA and TIM-3 on different GDs. Statistical analysis was performed using 2 way ANOVA. B. Effect of TIM-3 blockade on uNK cell population size. Percentage of CD3^-^ CD122^+^ NK1.1^-^ and DX5^-^ cell population in control and RMT3-23 treated pregnant CBA mice at GD 7.5, 10.5 and 12.5. There was no significant change in the number of uNK cells between control and treated groups. Data are presented as mean± SEM and are representative of at least 3 experiments. Statistical analysis was done by one-way analysis of variance (ANOVA) using Kruskal-Wallis test. C. Effect of TIM-3 blockade on uNK cell cytotoxicity. No significant change was observed in the cytotoxicity of uNK cells following treatment with RMT3-23. Data are presented as mean± SEM and are representative of at least 3 experiments. Statistical analysis was done using unpaired t test. D. Effect of TIM-3 blockade on Granzyme production by uNK cells. A small but significant increase in the production of Granzyme B was observed in the RMT3-23 treated uNK cells in comparison to control. Data are presented as mean± SEM and are representative of at least 3 experiments. Statistical analysis was done using unpaired t test.

Peripheral NK cells express TIM-3 on surface and TIM-3 expression is reported to regulate the cytotoxicity and IFN-γ secretion by the peripheral NK cells [14,15]. Over expression of TIM-3 on peripheral NK cell response in chronic viral infections is known to make them less responsive [9]. However, uterine NK cells differ from peripheral cells in both their surface receptor expression and function, and to our knowledge there are no reports of TIM-3 expression on uNK cells during gestation. We have previously observed that TIM-3 is expressed at the FMI and also on the uterine macrophage population [12]. So we first determined if TIM-3 is expressed on the uNK cells and the kinetics of TIM-3 expression during gestation, particularly between GD 7.5 and 12.5, the gestation period when uNK cells accumulate in large numbers in the uterus. Uterine NK cells are reactive to the *Dolichos biflores* agglutinin (DBA) and are the only uterine lymphocyte population that binds to DBA [19]. Peripheral NK cell population from either the CBA or C57BL/6 strain does not bind to DBA[20]. DBA expression is increased on the CD3^-^ CD122^+^ NK1.1^-^ DX5^-^ uNK cell population in pregnant CBA mice. We measured TIM-3 expression and compared it to the expression of DBA, the marker of uNK cells that is also up regulated between GD7.5 and 12.5 on CD3^-^ CD122^+^ NK1.1^-^ DX5^-^ NK cells. The uNK cells were isolated from the uteri of pregnant CBA mice that were allogeneically mated with C57BL/6 males at three different time points, GD 7.5, 10.5 and 12.5. We observed that TIM-3 is expressed on uNK cells and the number of uNK cells expressing TIM-3 increases steadily from GD 7.5 to 12.5, which is very similar to the expression of DBA on these cells [Fig 3A]. Our data show that uNK cells up regulate TIM-3 during mid gestation, the time period when the uNK cells play a key role in regulating the local environment at the FMI.

### Effect of TIM-3 blockade on uNK cell population

To further understand the role of TIM-3 expression on uNK cells and how it affects the feto-maternal tolerance, we studied the effect of TIM-3 blockade on the CD3^-^ CD122^+^ NK1.1^-^DX5^-^ DBA^+^ cell population. We used DBA as the marker to determine the uNK cell population in the allogeneically mated (CBA female x C57BL/6 male) female CBA mice as these lack NK1.1 reactivity, and observed that the DX5+ cells were also DBA negative. Yadi et al., have also reported similar observations previously[21]. We used mAb RMT3-23 to block TIM-3 and observed the effect on the CD3^-^ CD122^+^ NK1.1^-^DX5^-^ DBA^+^ cell population in the uteri at GD 7.5, 10.5 and 12.5. Blockade of TIM-3 did not have any effect on the population size of CD3^-^ CD122^+^ NK1.1^-^DX5^-^ DBA^+^ cells between GD 7.5 and 12.5. There was no significant change in the number of NK1.1^-^DX5^-^ DBA^+^ cells in the uterus between GD 7.5 and 12.5 following TIM-3 blockade [Fig 3B].

### Effect of TIM-3 blockade on uNK cell cytotoxicity

We next determined the effect of TIM-3 blockade on the cytotoxic ability of the uNK cells. It is already known that the cytolytic ability of the uNK cells is low and can be inhibited by the interaction of uNK cells with non-classical MHC molecules [22,23]. On the other hand, under certain stress induced circumstances uNK cells are also known to revert back to their conventional cytotoxic mode and cause fetal loss [24,25,26]. Therefore, we determined both the cytotoxic ability and granzyme B production by the CD3^-^ NK1.1^-^ DX5^-^ DBA^+^ cell population that was isolated from allogeneically mated (CBA female x C57BL/6 male) female CBA mice. Cytotoxicity was determined by the percent killing of YAC-1 target cells by uNK cells following treatment with RMT3-23 and the control group. Treatment with RMT3-23 did not result in any change in the cytotoxic ability of the uNK cells [Fig 3C] although a small but significant increase in granzyme B production (p = 0.0184) was observed following treatment with RMT3-23 [Fig 3D].

### Modified uNK cell phenotype following treatment with RMT3-23

We next explored the effect of TIM-3 blockade on the phenotype of uterine NK cells. In the experiments that follow we considered the NK1.1^-^ DX5^-^ DBA^+^ cells to be the uNK population from the pregnant CBA mice that were allogeneically mated to C57BL/6 males.

Blockade of TIM-3 has a distinct effect on the surface expression of a number of receptors on the uNK cells. First we looked at the expression of CD11b and CD27 on the NK1.1^-^ DX5^-^ DBA^+^ cells. The CD11b^+^ CD27^lo^ NK cells are known to reside in non-lymphoid tissue and are functionally distinct in their cytotoxic ability and cytokine production and show a distinct inhibitory receptor expression [27]. The NK1.1^-^ DX5^-^ DBA^+^ cells were CD27^lo^ and CD11b^+^ and there was no change in the expression following blockade of TIM-3 (Data not shown).

NKp46 is a NK cell marker conserved across species [28] and is expressed in low levels in uNK cells [21]. NKG2D is another receptor expressed by all NK cells [29,30]. We determined the expression of these two markers on the NK1.1^-^ DX5^-^ DBA^+^ uNK cells following TIM-3 blockade. Our data shows no change in the expression of either NKp46 or NKG2D following treatment with RMT3-23 (Data not shown).

**Fig 4:**
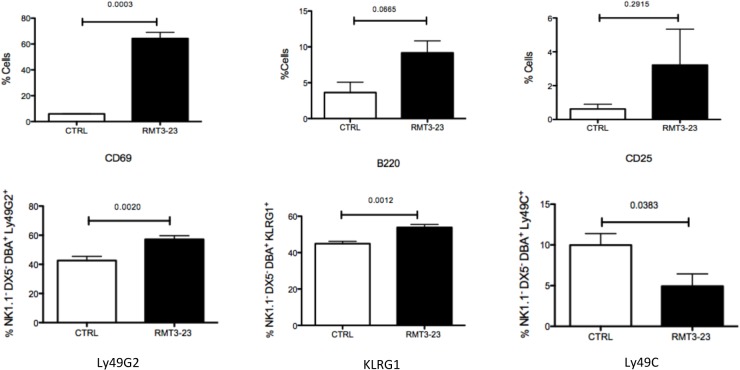
Effect of RMT3-23 treatment on expression of activation markers and inhibitory surface receptors on uNK cells. Treatment with RMT3-23 significantly upregulates the expression of CD69 on NK1.1^-^ DX5^-^ DBA^+^ cell population. A similar trend of increased expression was also observed in case of B220 and CD25 levels after treatment with RMT3-23 although the data were not statistically significant. RMT3-23 treatment also significantly down regulates Ly49C expression and up regulates KLRG1 and Ly49G2 on NK1.1^-^ DX5^-^ DBA^+^ cells. Data are presented as mean± SEM and are representative of at least 3 experiments. Statistical analysis was done using unpaired t test.

We next analyzed the expression of various activating and inhibitory receptors on the NK1.1^-^ DX5^-^ DBA^+^ population from the uteri of mice. Following treatment with RMT3-23, expression of CD69 was increased (p = 0.0003) [Fig 4], a significant decrease in Ly49C (p = 0.0383) expression was observed, whereas the expression of Ly49G2 (p = 0.0020) and KLRG1(p = 0.0012) was significantly increased [Fig 4]. We did not observe any change in the expression of B220 and CD25 following RMT3-23 treatment [Fig 4].

Although TIM-3 blockade did not change the percentage of uNK cells or their cytotoxicity, we observed a selective change in the expression of surface receptors, both activating and inhibitory in nature, that regulate the uNK cells.

### Distinct modulation of uNK cells following blockade of TIM-3

**Fig 5:**
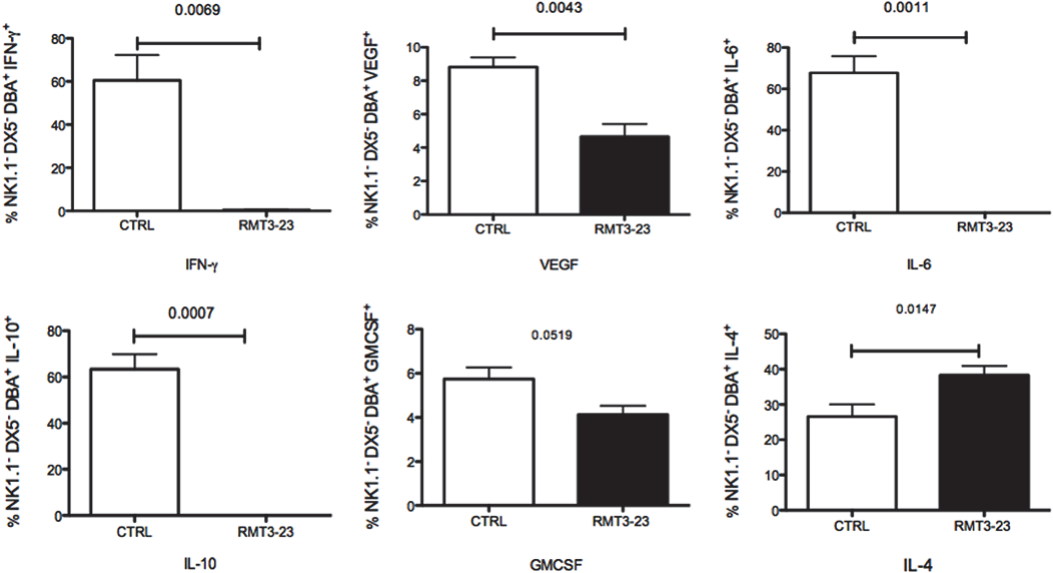
Effect of RMT3-23 treatment on cytokine production by uNK cells. Treatment with RMT3-23 significantly down regulated cytokine production by NK1.1^-^ DX5^-^ DBA^+^ cell population. A significant decrease in the population of IFN, IL-6, IL-10, VEGF and GMCSF producing NK1.1^-^ DX5^-^ DBA^+^ cells was observed after treatment with RMT3-23 in comparison to Control group, whereas IL-4 production was significantly increased. Data are presented as mean± SEM and are representative of at least 3 experiments. Statistical analysis was done using unpaired t test.

We next determined the effect of TIM-3 blockade on various cytokines produced by uNK cells from allogeneically mated (CBA female x C57BL/6 male) female CBA mice. Our data showed significant changes in cytokine productions by uNK cells after treatment with RMT3-23. Both IFN-γ (p = 0.0069) and VEGF (p = 0.0043) productions were significantly reduced in the RMT3-23 treated DBA^+^ uNK cell population in comparison to the control [Fig 5]. Treatment with RMT3-23 also resulted in a significant decrease in IL-6 (p = 0.0011) production by the uNK cells. A significant decrease in IL-10 production (0.0007), [Fig 5] a cytokine associated with tolerance induction, was also observed following TIM-3 blockade. We also observed a decrease in GMCSF production by uNK cells but the data was not statistically significant. However, we observed an increase in IL-4 (p = 0.0147) production following RMT3-23 treatment by the uterine NK cells [Fig 5]. We did not observe any changes in TGF-β and TNF production by the uNK cells following treatment with RMT3-23 (data not shown). Our results suggest that blockade of TIM-3 results in a significant decrease in the production of a number of cytokines by the uNK cell that are essential for a successful pregnancy.

### TIM-3 blockade affects GMCSF production in the placenta

**Fig 6:**
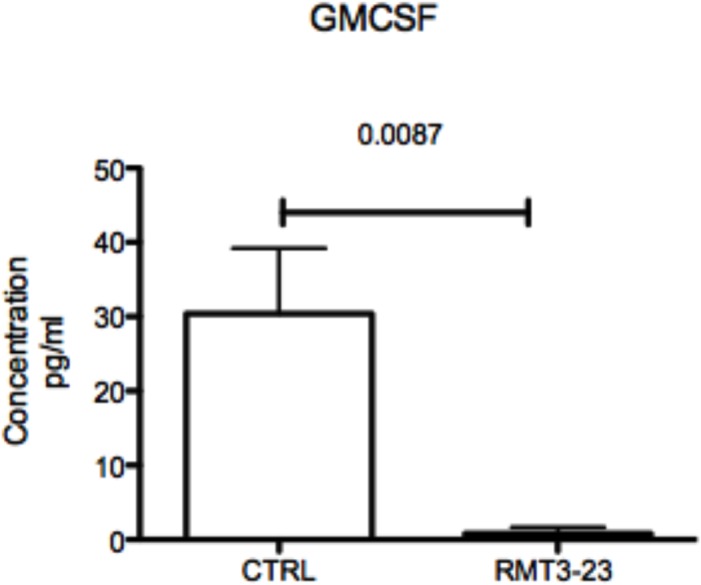
GMCSF concentration in the placenta of CBA/CaJ mice at GD 12.5 following TIM-3 blockade. A significant decrease in the GMCSF concentration was observed in the placenta of RMT3-23 treated mice in comparison to control. Data are presented as mean± SEM and are representative of at least 3 experiments. Statistical analysis was done using unpaired t test.

Our previous data shown in Fig 5 illustrates that RMT3-23 treatment decreases GMCSF production by uNK cells. Uterine NK cells however, are not the primary cells producing GMCSF at the FMI. GMCSF is produced mostly by uterine epithelial cells and during pregnancy synthesis of GMCSF persists in the placenta. So we determined the level of GMCSF production in the placenta of allogeneically mated (CBA female x C57BL/6 male) female CBA mice following TIM-3 blockade. We observed that following treatment with RMT3-23 the concentration of GMCSF in the placenta decreases significantly (p = 0.0087) in the treated group in comparison to that of the control mice [Fig 6]. GMCSF is essential for the expansion and immunosuppressive potential of myeloid derived suppressor cells (MDSC), a heterogeneous population of immature myeloid cells that have potent immunosuppressive properties. The MDSC are present at the FMI and contribute to the tolerant environment as we have observed and reported previously [12]. Along with GMCSF, IL-6 is also reported to be important in promoting the generation of MDSC and switch the microenvironment towards immunosuppression. Our data showed that TIM-3 blockade significantly decreases both GMCSF [Fig 6] and IL-6 [Fig 5] at the FMI.

### Plasticity of uNK cells and uMDSCs at the feto maternal interface

To further understand the role of GMCSF on uNK cells and uMDSCs, we flow sorted uNK cells between GD10.5 and 12.5 from female CBA mice allogeneically mated with C57BL/6 males (NK1.1^-^ DX5^-^ DBA^+^) and female C57BL/6 mice allogeneically mated with CBA males (NK1.1^+^ DX5^-^ DBA^+^). The two different uNK cell populations were cultured in vitro in presence of GMCSF (20ng/ml) with or without IL-2 (20ng/ml) for 5 to 7 day. These cells were then analysed using a flow cytometer to detect the change in the respective uNK (NK1.1^-^ DX5^-^ DBA^+^ or NK1.1^+^ DX5^-^ DBA^+^) and uMDSC (CD11b^+^ Ly6C^hi^ Ly6G^lo^ and CD11b^+^ Ly6C^lo^ Ly6G^hi^) cell populations.

**Fig 7:**
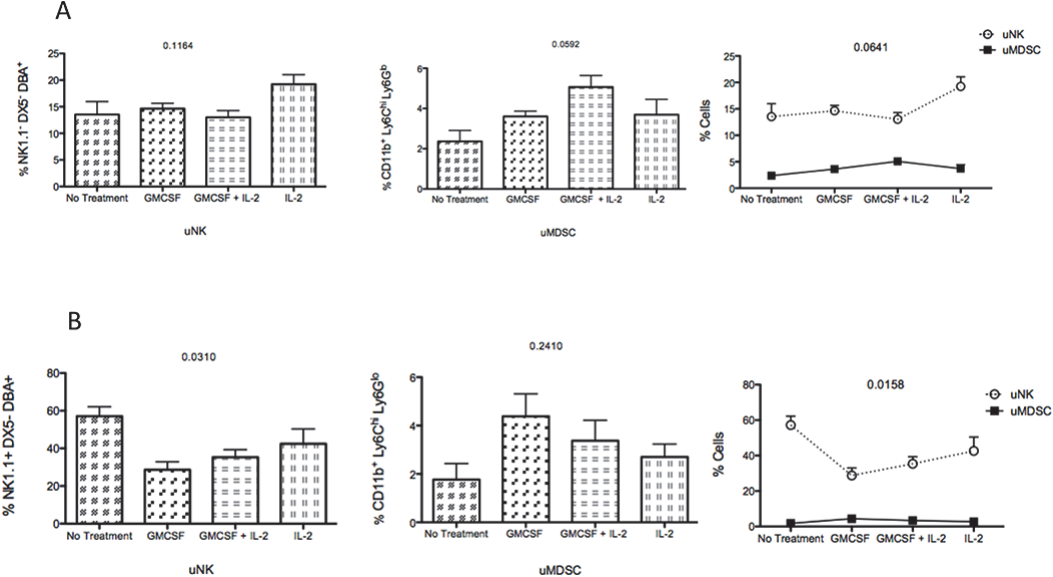
Effect of GM-CSF on the uNK cells and uMDSC from pregnant mice. A. CBA/CaJ female mice were mated with C57BL/6 allogeneic male mice. Uterine NK cells were flow sorted to obtain a NK1.1^-^ DX5^-^ DBA^+^ cell population that was incubated with GMCSF (20ng/ml) ± IL-2 (20ng/ml) for 5–7 days. Expression of uNK and CD11b^+^Ly6G^hi^Ly6C^lo^ and CD11b^+^Ly6G^lo^Ly6C^hi^ cell populations were analyzed using a flow cytometer. GMCSF treatment resulted in no significant change in the NK1.1^-^ DX5^-^ DBA^+^ cell population from the CBA mice (p = 0.1164). However, an increase of the CD11b^+^Ly6G^lo^Ly6C^hi^ population was observed (p = 0.0592). An inverse correlation was observed between the uNK cells and uMDSC in presence of both GMCSF and IL-2 treatment but was not statistically significant. B. C57BL/6 female mice were mated with allogeneic CBA/CaJ male mice. Uterine NK cells were flow sorted to obtain a NK1.1^+^ DX5^-^ DBA^+^ cell population that was incubated with GMCSF (20ng/ml) ± IL-2 (20ng/ml) for 5–7 days. Expression of uNK and CD11b^+^Ly6G^hi^Ly6C^lo^ and CD11b^+^Ly6G^lo^Ly6C^hi^ cell populations were analyzed using a flow cytometer. GMCSF treatment resulted in a significant change in the NK1.1^+^ DX5^-^ DBA^+^ cell population from the C57BL/6 female mice (p = 0.0310). However, the change in the CD11b^+^Ly6G^lo^Ly6C^hi^ population was not significant (p = 0.2410). The effect of GMCSF was more pronounced in the uNK cells from the C57BL/6 female mice and a significant inverse correlation between uNK and uMDSC was also observed (p = 0.0158). Data are presented as mean± SEM and are representative of at least 3 experiments. Statistical analysis was done by one-way analysis of variance (ANOVA) using Kruskal-Wallis test and two way ANOVA.

Fig 7A shows the effect of GMCSF on the NK1.1^-^ DX5^-^ DBA^+^ cells from the pregnant CBA mice. GMCSF treatment resulted in no significant change in the NK1.1^-^ DX5^-^ DBA^+^ cell population from the CBA mice (p = 0.1164). In case of uNK cells from female CBA mice GMCSF treatment alone did not have any visible effect on the uNK cells. IL-2 treatment alone resulted in a small but statistically insignificant increase in the uNK cells. The small change resulted from IL-2 treatment was completely abrogated in the presence of GMCSF. However, an increase in the CD11b^+^Ly6G^lo^Ly6C^hi^ population was observed that was not statistically significant (p = 0.0592). GMCSF treatment alone as well as in combination with IL-2 resulted in an increase in the CD11b^+^Ly6G^lo^Ly6C^hi^ population. We also observed an inverse correlation between the uNK cells and uMDSC following treatment with GMCSF as well as IL-2 (p = 0.0641) that was not statistically significant.

The effect of GMCSF was more pronounced in the uNK cells from C57BL/6 females [Fig 7B] and a significant decrease in uNK cell population was observed following GMCSF treatment (p = 0.0310). On the other hand the change in the CD11b^+^Ly6G^lo^Ly6C^hi^ population was not significant (p = 0.2410). A significant inverse correlation (Interaction p = 0.0158, Column factor<0.0001, Row factor 0.0482) was observed between the uNK and uMDSC cell populations isolated from the C57BL/6 females in response to GMCSF and IL-2.

**Fig 8:**
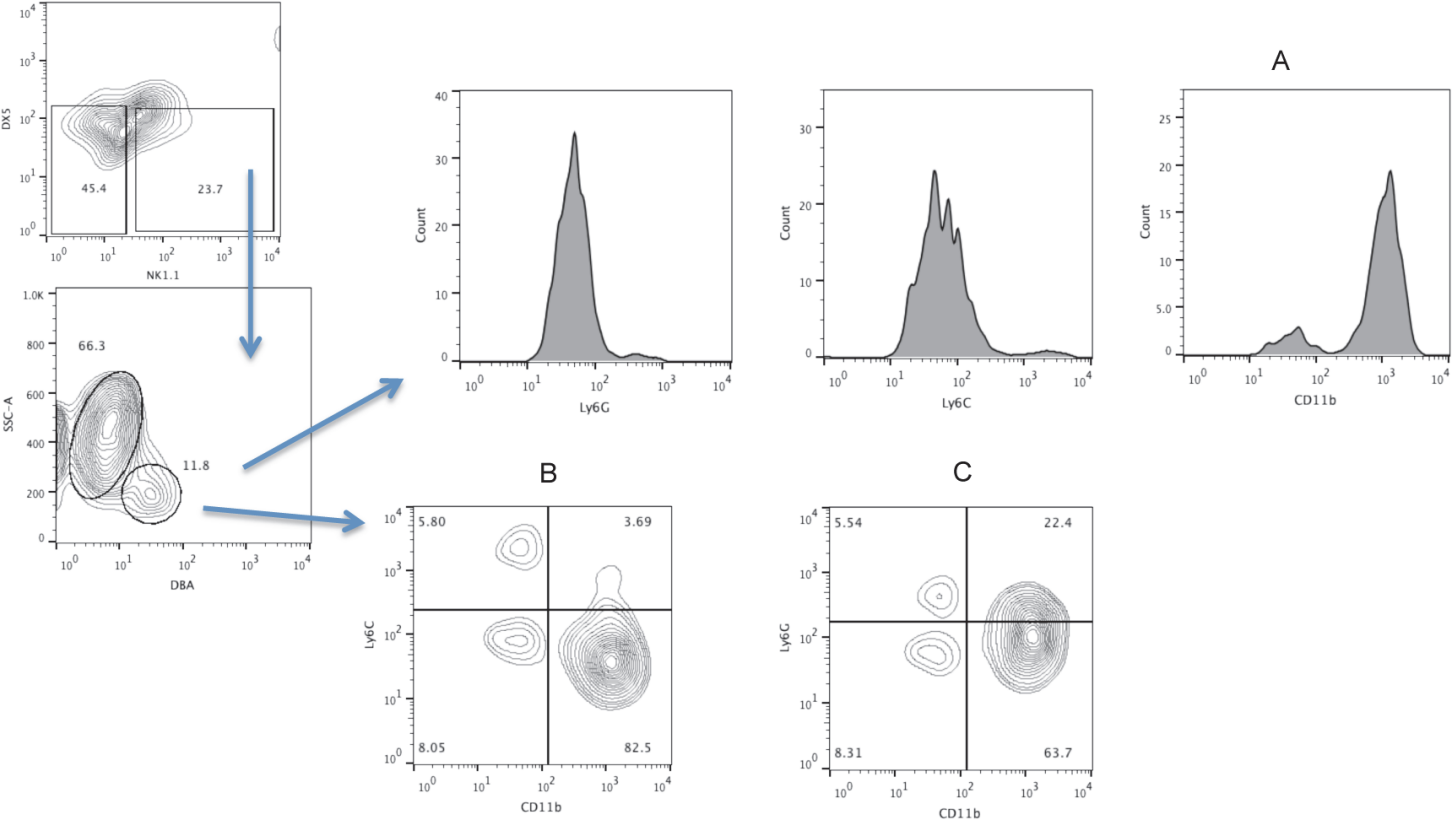
Expression of CD11b, Ly6C and Ly6G on uNK cells from CBA mice. Female CBA mice were allogeneically mated toC57BL/6 males and uterine lymphocytes were isolated between GD 10.5 to 12.5. Expression of CD11b, Ly6C and Ly6G was determined on the NK1.1^-^ DX5^-^DBA^+^ cell population. Data are representative of at least 4 experiments.

To further understand the plasticity between the uNK and uMDSC cell populations, we determined if the conventional MDSC markers are expressed on the uNK cells. MDSC are classified by the expression of Ly6G and Ly6C molecules into two different subsets in mouse namely, CD11b^+^Ly6C^lo^Ly6G^hi^ and CD11b^+^Ly6C^hi^Ly6G^lo^. We have mentioned previously that the uNK cells express CD11b, which is also a marker for MDSC. We next determined the expression of Ly6C and Ly6G on the NK1.1^-^DX5^-^DBA^+^ uNK cell population from allogeneically mated pregnant CBA mice. Our data show that all the three MDSC markers are expressed on the uNK cells [Fig 8].

**Fig 9:**
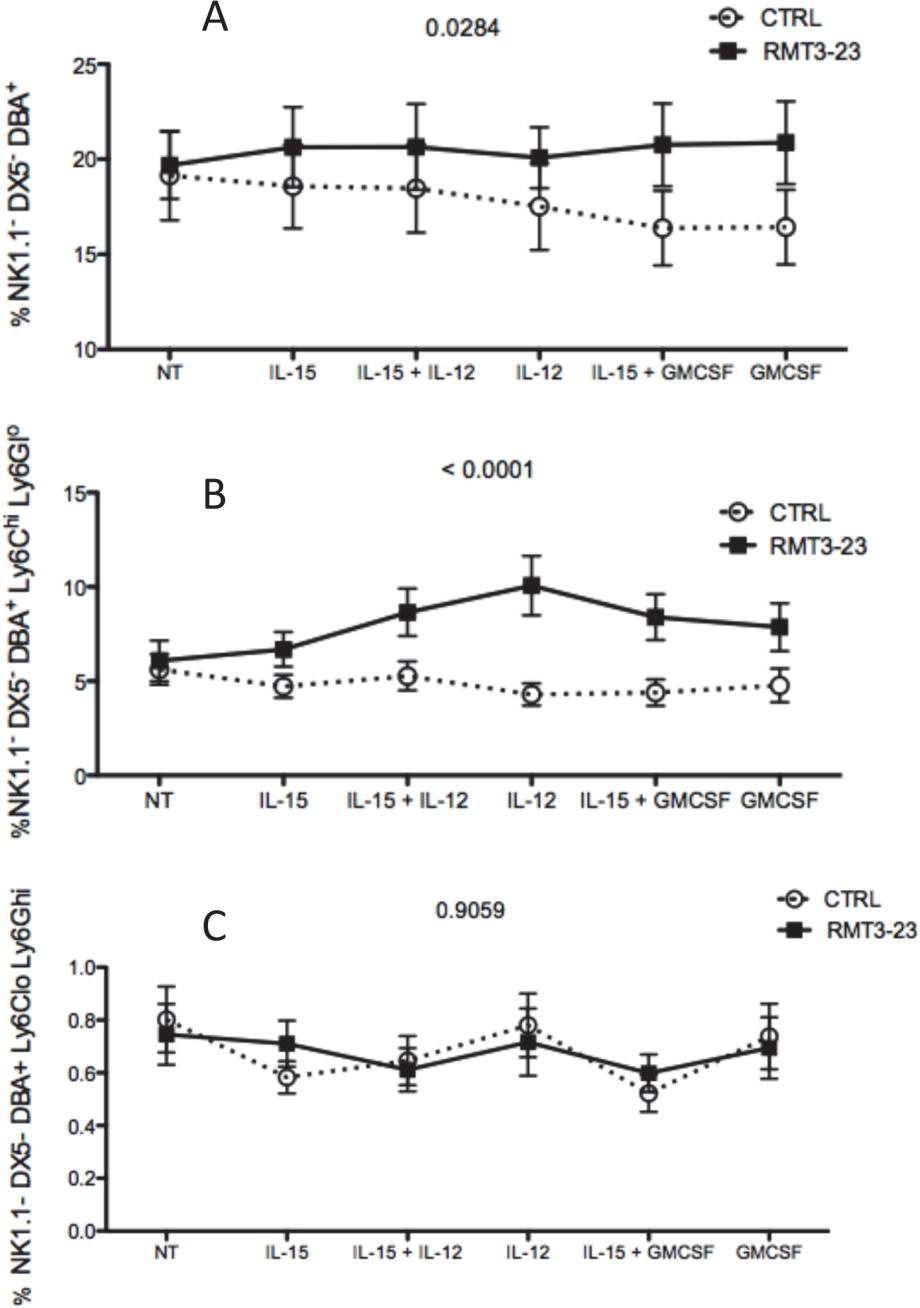
Effect of GM-CSF and IL-15 on the uNK cells from pregnant CBA mice. CBA/CaJ female mice were mated with C57BL/6 allogeneic male mice. Uterine lymphocytes were isolated from Control and RMT3-23 treated mice and cultured with IL-15, IL-15+IL-12, IL-12, IL-15+GMCSF and GMCSF only for 7days in vitro in a 37°C humidified CO2 incubator. At the end of the incubation period the percentage of 3 different cell populations were determined by flow cytometry. A. RMT3-23 treated uNK cells proliferated more in response to IL-12 (p = 0.0284). B. This effect was more pronounced in the uNK cells expressing the MDSC markers (p<0.0001).

Our next experiment was aimed at finding if TIM-3 blockade affects the uNK cells in presence of cytokine treatment. Uterine lymphocytes were isolated from control and RMT3-23 treated allogeneically mated (CBA female x C57BL/6 male) pregnant CBA mice between GD 10.5 and 12.5 and incubated with various cytokine combinations (IL-15, IL-15+IL-12, IL-12, IL-15+GMCSF, GMCSF). After 7 days we determined the percentage of three cell populations, uNK cells (NK1.1^-^DX5^-^DBA^+^) and uNK cells expressing the MDSC markers (NK1.1^-^DX5^-^DBA^+^Ly6C^hi^Ly6G^lo^ and NK1.1^-^DX5^-^DBA^+^Ly6C^lo^Ly6G^hi^). We observed RMT3-23 treated cells proliferated more than the control cells in response to the cytokine treatment (Fig 9). RMT3-23 treated uNK cells increased in number in response to GMCSF and GMCSF+IL-15 treatment (p<0.05), whereas the number of uNK cells from control mice decreased in response to the same treatment (Fig 9A, p= 0.0284). The number of NK1.1^-^DX5^-^DBA^+^Ly6C^hi^Ly6G^lo^ cells increased in response to IL-12 treatment in presence of RMT3-23, whereas the control NK1.1^-^DX5^-^DBA^+^Ly6C^hi^Ly6G^lo^ cell number decreased (p<0.001). Similarly in response to GMCSF treatment, RMT3-23 treated NK1.1^-^DX5^-^DBA^+^Ly6C^hi^Ly6G^lo^ cell number went down whereas, the control NK1.1^-^DX5^-^DBA^+^Ly6C^hi^Ly6G^lo^ cell number increased (Fig 9B, p<0.0001). We did not see any significant difference between the control and RMT3-23 treated NK1.1^-^DX5^-^DBA^+^Ly6C^lo^Ly6G^hi^ cell populations (Fig 9C, p = 0.9059).

### TIM-3 blockade affects the suppressive potential of uterine myeloid derived suppressor cells (MDSC)

**Fig 10:**
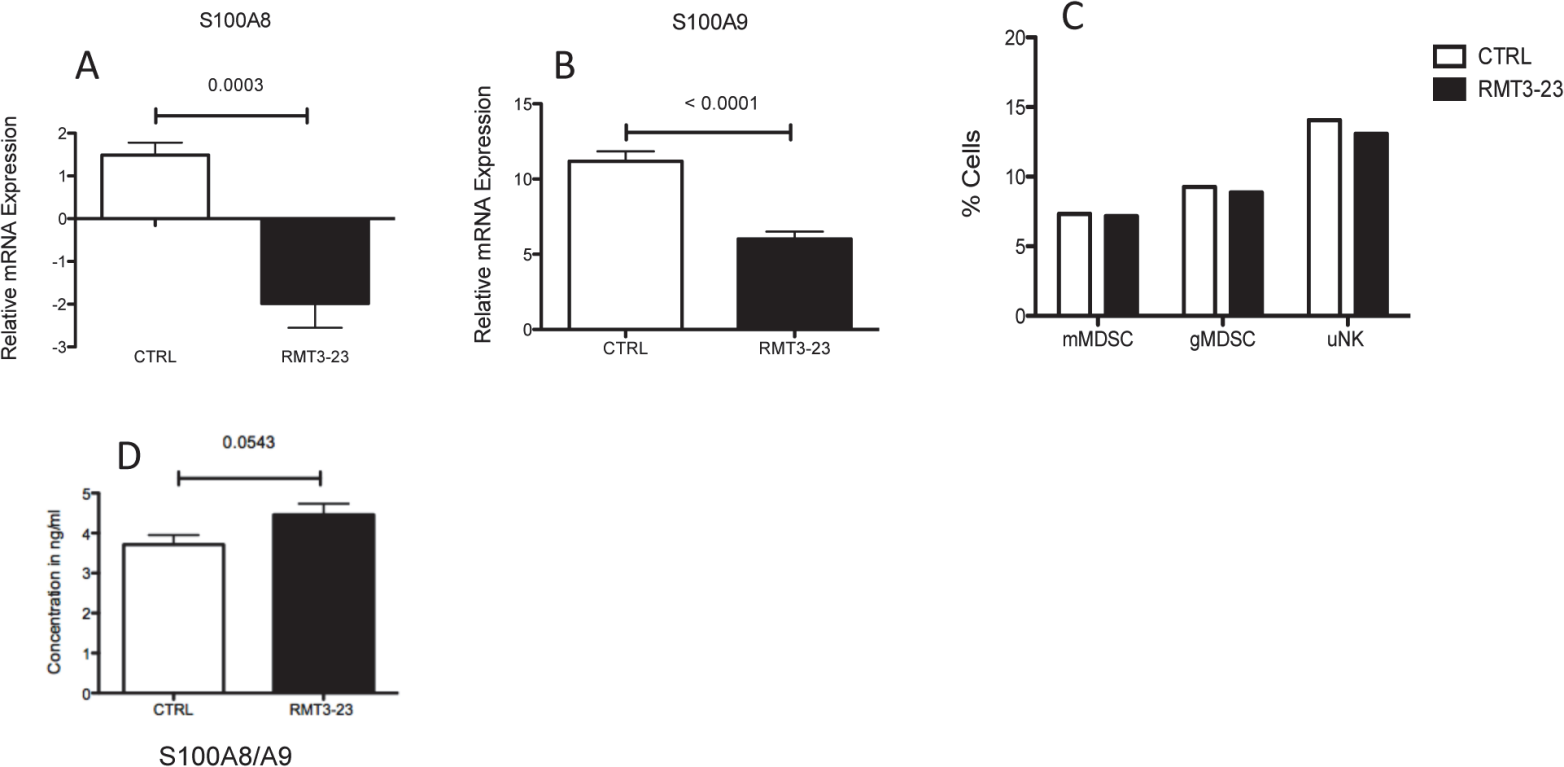
Effect of TIM-3 blockade on uterine MDSC population. Treatment with RMT3-23 did not change the size of the MDSC population in the uteri of pregnant CBA mice (C) although a significant decrease in the proinflammatory S100A8 (p = 0.0003) (A) and A9 (p<0.0001) (B) proteins in the placentae was observed. No change was observed in the concentration of the S100A8/A9 proteins in the RMT3-23 treated mice sera (D). Data are presented as mean± SEM and are representative of at least 3 experiments. Statistical analysis was done using unpaired t test.

MDSC are induced by the proinflammatory S100A8/A9 proteins [31]. Therefore, we also determined the expression levels of S100A8/A9 proinflammatory proteins in the placentae and sera of mice following RMT3-23 treatment. A significant decrease in the mRNA levels of both S100A8 (p = 0.0003) and A9 (p<0.0001) proteins were observed in the placentae of RMT3-23 treated female CBA mice that were allogeneically mated to C57BL/6 males, in comparison to the control (Fig 10A and 10B) whereas there was no significant difference in the concentration of S100A8/A9 proteins in the sera of RMT3-23 treated mice in comparison to control (Fig 10D). However, we did not see any significant difference in the size of the MDSC populations following RMT3-23 treatment (Fig 10C). This was similar to our previous observation that following treatment with RMT3-23 the size of the NK cell population does not change significantly.

**Fig 11:**
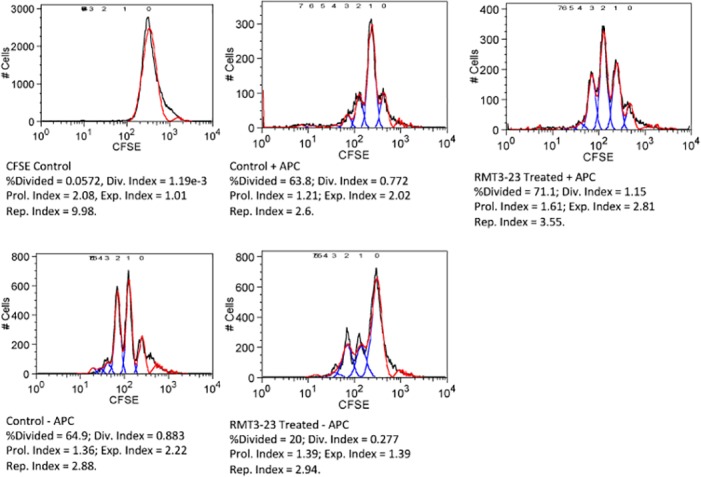
Effect of TIM-3 blockade on the suppressive function of uterine MDSC. Uterine MDSC were flow sorted from Control and RMT3-23 treated pregnant mice between GD10.5 to 12.5. The uMDSC were co cultured with CFSE stained CD4+ T cells from OT II mouse in presence of allogeneic CD90^-^APC and OVA_323-339_ peptide. Following 72h. of incubation cell proliferation was determined by CFSE dilution using a flow cytometer. Data represents 2 experiments.

To further understand the effect of TIM-3 blockade on the uterine MDSC, we determined the suppressive effect of uterine MDSC on T cell proliferation. Uterine MDSC (CD11b^+^ Ly6C^+^ Ly6G^+^) were flow sorted from Control and RTM-23 treated CBA mice, that were allogeneically mated to C57BL/6 males, between GD 10.5 and 12.5 and cultured in vitro with CD4^+^ T cells from OT II mouse that are specific for the OVA_323-339_ peptide in the presence of allogeneic CD90^-^ APCs from C57BL/6 mice. The control uMDSC were able to suppress the proliferation of antigen specific CD4^+^T cells (Replication Index 2.6). However, the RMT3-23 treated uMDSC were less able to suppress the antigen specific proliferation of CD4^+^ T cells (Replication Index 3.55) [Fig 11]. The data in Fig 11 represents 2 experiments only. Interestingly, we did not observe a similar suppressive activity of the uMDSC on CD8+ T cells (data not shown).

## Discussion

In the present study we have demonstrated that TIM-3 regulates the unique receptor phenotype and cytokine production by uNK cells, and blockade of TIM-3 decreases the suppressive function of uterine MDSC. It is evident from our data that TIM-3 plays a regulatory role, and contributes to the maintenance of tolerance at the FMI by affecting these two cell types of the innate immune system.

NK cells are the major lymphocyte population in the feto-maternal interface, possess a unique receptor phenotype, lack cytotoxicity, and produce various cytokines that are essential for the survival of the fetus. Recently, TIM-3 was shown to play an important role in the regulation of innate immune cells like macrophages and NK cells [32]. We have previously shown that TIM-3 plays an important role in the phagocytic potential of uterine macrophages. Blockade of TIM-3 results in decreased phagocytosis by uMacs and apoptotic bodies accumulate at the FMI, thus altering the fine balance between immunity and tolerance that exists at the interface [12]. Uterine macrophages are the second most abundant lymphocyte population at the FMI, the first one being the uNK cells and TIM-3 is reported to regulate NK cells too [32]. Expression of TIM-3 is reported to suppress cell-mediated cytotoxicity [14]. Increased TIM-3 expression leads to increased IFN-γ production by NK cells [15] and to dysregulated NK cell function. During gestation, the uNK cells play a significant role in establishment and maintenance of pregnancy related vascularization as well as tolerance to the fetus.

Our results showed that the uNK cells up regulate TIM-3 expression between GD 7.5 and 12.5 similar to the expression of DBA, a marker that is up regulated by the uNK cells during gestation. Blockade of TIM-3 resulted in fetal loss [12] and also decreased production of IL-15 and IL-9 in the placentae, the cytokines essential for NK cell survival and development, indicating an unfavorable environment for uNK cells at the FMI. The cytokine IL-15 is essential for the survival of the uNK cells [33] at the FMI and is secreted abundantly by the placenta [34] Mice lacking a functional IL-15 gene show complete absence of uNK cells and these cells can be restored with exogenous IL-15 [35]. Barber and Pollard also report that IL-15 is required for the differentiation of NK cells during pregnancy[35]. In our experiments TIM-3 blockade results in a decreased production of IL-15, not a complete absence of IL-15, which is probably not enough to affect the uNK cell percentage. Instead, our data showed a distinct change in the unique surface receptor phenotype of the uNK cells and also a significant decrease in the various cytokines produced by these cells following TIM-3 blockade.

Uterine NK cells have a unique phenotype and maintain a dynamic balance between activating and inhibitory receptors. The presence or absence of appropriate inhibitory receptors is a deciding factor in the recognition of fetal trophoblast cells by maternal uNK cells. Lack of inhibitory receptors has also been associated with fetal loss in humans [36,37]. The unique receptor repertoire of uNK cells includes the inhibitory receptors Ly49C and Ly49G2. The Ly49C receptor on the maternal (CBA) uNK cell recognizes the paternal (C57BL/6) H-2k^b^ on the trophoblast cells and induces tolerance. Ly49 receptor knock out mice are reported to have pregnancy defects [38]. Our data showed a decrease in the expression of Ly49C receptors on the uNK cells following TIM-3 blockade, which most probably affects the interaction between uNK cells and trophoblasts.

Interestingly, expression of two other inhibitory receptors, Ly49G2 and KLRG1 were increased following TIM-3 blockade and also the expression of early marker of activation, CD69, was increased. An inverse correlation exists between CD69 and KLRG1 expression on uNK cells during gestation as previously reported by Croy et al., [39]. TIM-3 blockade resulted in disruption of this inverse correlation. This aberrant expression possibly contributed to a lack of optimal function of uNK cells at the FMI.

Significant down regulation in the expression of inhibitory receptors like Ly49C on uNK cells together with up regulation of activation markers like CD69 following TIM-3 blockade indicates a change in the expression pattern of the receptors phenotype that may alter the recognition of self by uNK cells and play a role in fetal loss.

In addition to affecting the receptor repertoire, TIM-3 blockade also results in a distinct modulation of the cytokine production by uNK cells. Uterine NK cells are cytokine producers and these cytokines play an important role in pregnancy associated vascularization and maintenance of a tolerant environment at the FMI. Production of both proangiogenic and immunosuppressive cytokines by uNK cells were decreased following TIM-3 blockade. TIM-3 blockade significantly decreased the production of IFN-γ and VEGF, the two major cytokines essential for pregnancy-associated vascularization. Significant reduction in VEGF and IFN-γ production suggests an inhibitory effect on vascular remodeling. TIM-3 blockade also resulted in significant decrease in IL-10 production by uNK cells. IL-10 is an inhibitory cytokine responsible for maintenance of a tolerant environment at the FMI. The reduced level of IL-10 production creates an imbalance in the tightly controlled tolerant microenvironment at the FMI.

The reduction in IL-6 and GMCSF production adds to this imbalance by affecting the MDSC that also help maintain the tolerance. The decrease in the proinflammatory S100 proteins at the mRNA level following TIM-3 blockade also indicates an adverse effect on the uterine MDSC population. The S100A8/A9 proteins serve as an autocrine feedback loop that sustains the MDSC and contributes to the maintenance of tolerance [31]. Although we did not observe a decrease in the levels of MDSC population following TIM-3 blockade, a significant decrease in the suppressive potential of these cells towards CD4^+^ T cells were observed indicating a disruption of the fine balance between tolerance and immunity at the FMI. An increase in the number of T helper cells due to a decreased suppressive function of MDSCs following Tim-3 blockade at the FMI might creates a bias towards a proinflammatory environment and inflammation in the late stages of gestation. This is significant considering that inflammation in the late stages of gestation is one of the factors associated with fetal loss [40].

These results demonstrate that TIM-3 modulates the uNK cells at the FMI and plays a role in maintaining the tolerance that is necessary for a successful pregnancy.

We have shown previously that TIM-3 blockade results in decreased phagocytic ability of the uterine macrophages [12]; and on the other hand increases the apoptosis of CD4+ CD25+ T cells (unpublished data) suggesting that TIM-3 blockade affects a variety of cells at the FMI that are directly or indirectly involved in the maintenance of tolerance and prevention of fetal loss. Our current and previously published data show that the two major uterine lymphocyte populations, uMacs and uNK cells, are affected directly by TIM-3 blockade. It is also possible that both the uMacs and uNK cells affect each other in turn. In vivo depletion of specific uterine lymphocyte population may shed more light into this.

TIM-3 is expressed on another subset of innate immune cells namely the MDSC at the FMI during mid gestation and blockade of TIM-3 results in activation of the uMDSC [12]. However our present study shows that the suppressive potential of the uMDSC is decreased following TIM-3 blockade.

A highly interesting aspect of our study is the plasticity between uNK and uMDSC cell populations. The uNK cells express the phenotypic markers of uMDSC and it has been shown before that in presence of GMCSF, NK cells are capable of converting into cell type similar to MDSC [41]. We also observe a similar phenomenon at the FMI. The uNK cells express low levels of MDSC surface markers and in presence of GMCSF; the expression of Ly6C on the uNK cells is increased. Although it is reported that NK cells completely loose their NK markers and convert to MDSC in a tumor microenvironment, we did not see a similar phenomenon in our experiments instead we observed an increase in expression of MDSC surface marker on uNK cells. This increased expression of MDSC specific markers indicates a plasticity of uNK cells towards a MDSC phenotype that probably contributes to the immunosuppressive environment at the FMI. We surmise, the plasticity of uNK cells towards a MDSC phenotype, is a phenomenon observed in allogeneic matings and may not be seen in syngenic mice models of pregnancy due to the absence of allogeneic paternal antigens, but that remains to be proved.

The uNK cells are known to be unique in their surface receptor expression but it was very unusual to observe NK1.1 reactivity in the uNK population of CBA mouse. We have observed this in both syngeneic and allogeneic pregnancy models. The NK1.1 reactivity is not observed in the peripheral splenic NK cells. NK cells from CBA mouse strain do not react to the anti NK1.1 monoclonal antibody PK136. This is due to allelic divergence of the *Nkrp1b/c* gene in the CBA mouse strain that confers NK1.1 reactivity [18]. Both *Nkrp1b* and *Nkrp1c* gene products react with anti NK1.1 antibody PK136, and in BALB/c mice, that show no NK1.1 reactivity as CBA mice, these gene products are produced abundantly but lack reactivity due to divergence in protein sequences. Carlyle et al., in 2006 have shown that a single amino acid substitution could abolish NK1.1 reactivity, an effect that can also be reversed. The authors also suggested that reactivity between PK136 and NK1.1 epitope is not just the amino acid sequence but rather more conformational in nature [18]. More detailed experiments are required to understand the NK1.1 reactivity shown by the uNK cell population and to explain this unusual observation.

Collectively our data suggest that blockade of TIM-3 results in abrogation of the tolerant environment at the FMI by altering the function of both uNK cells and uMDSC. Following treatment with anti TIM-3 antibody, cytokine production by uNK cells is decreased and the local cytokine milieu changes from a pro angiogenic and pro immunosuppressive environment towards an inflammatory environment. In addition, the immunosuppressive potential of the uMDSC is also decreased following TIM-3 blockade. Decreased expression of immunosuppressive MDSC markers on uNK cells in absence of GMCSF indicates a less tolerant environment at the FMI. All of these changes produce an imbalance in the maintenance of a tolerant environment essential for the survival of the fetus and results in local inflammation and fetal rejection.
